# Genome Sequence of a Novel Iflavirus from the Leafhopper *Graminella nigrifrons*

**DOI:** 10.1128/genomeA.00323-15

**Published:** 2015-04-30

**Authors:** Yuting Chen, Sijun Liu, Bryony C. Bonning

**Affiliations:** Department of Entomology, Iowa State University, Ames, Iowa, USA

## Abstract

A novel iflavirus was discovered from the transcriptome of the leafhopper *Graminella nigrifrons*. The assembled virus genome has 9,700 nucleotides (nt) and encodes a 3,035-amino-acid polyprotein. Sanger sequencing was used to confirm the sequence encoding the polyprotein and indicated a genome length of 9,617 nt with a polyadenylated tail.

## GENOME ANNOUNCEMENT

The black-faced leafhopper, *Graminella nigrifrons* (Forbes), is one of the most abundant leafhoppers in the eastern United States (1). *G. nigrifons* vectors plant viruses including a semipersistently transmitted waikavirus, maize chlorotic dwarf virus (MCDV, *Sequiviridae*) (2, 3), and a persistently transmitted, emerging plant rhabdovirus, maize fine streak virus (MFSV, *Rhabdoviridae*) (4, 5). Control of the insect vector is a key approach for management of plant viral disease. Hence, in addition to use of chemical insecticides (6), novel control strategies, such as use of insect viral agents for vector management, is of interest.

The *G. nigrifrons* transcriptome was obtained from the NCBI GenBank short-read archive (accession no. SRP013390.3) (7). The Illumina reads were *de novo* assembled using Trinity (8), and the resulting contigs (>200 nt) were annotated by BLAST analysis against a local NCBI nonredundant (nr) protein database. Putative viral sequences were extracted for further analysis. A novel positive-sense single-stranded RNA (+ssRNA) virus sequence was identified. The polyprotein encoded by this sequence had 36% amino acid sequence identity to that of slow bee paralysis virus (accession no. AD146683.1). The proposed name for this virus is *Graminella nigrifrons virus 1* (GNV1).

The assembled GNV1 genome is 9,700 nucleotides (nt), including a 324-nt 5′ untranslated region (UTR) and a 271-nt 3′ UTR. The genome has one open reading frame (ORF) that encodes a 3,035-amino-acid polyprotein, which is typical for an iflavirus. Four conserved domains for viral structural proteins were identified at the N terminus: two rhv_like (accession no. cd00205, located at amino acids 203 to 384 and 515 to 663), one Rhv (pfam00073, 464 to 632), and one CRPV_capsid (pfam08762, 874 to 1,087). Three conserved domains for viral nonstructural proteins were located at the C terminus: RNA_helicase (pfam00910, 1,419 to 1,525); Peptidase_C3 (pfam00548, 2,344 to 2,438), and RdRP_1 (pfam00680, 2,503 to 3,026). The RdRP_1 domain is also homologous to the RNA_dep_RNAP domain (cd01699, 2,650 to 2,945). Phylogenetic analysis revealed that GNV1 is closely related to slow bee paralysis virus.

To confirm the GNV1 genome sequence, reverse transcription-PCR (RT-PCR) with overlapping primers and rapid amplification of cDNA ends (RACE), followed by Sanger sequencing were used. Viral RNA was isolated from a *G. nigrifrons* laboratory colony. The first-strand cDNA was synthesized using ProtoScript II reverse transcriptase (New England Biolabs) or the SMARTer RACE cDNA amplification kit (Clontech). The resulting viral genome is 101-nt shorter than the assembled sequence at the 5′ UTR, 14 nt differed at the 3′ UTR, and a 3′ polyadenylated tail was identified. In addition, the confirmed genome has nine nucleotides less than that of the assembled sequence in the coding region. Hence, the confirmed genome encodes a polyprotein of 3,032 amino acids. Based on the assembled sequence and Sanger sequencing results, GNV1 is AT rich, with an A+T content of 61.64%.

The transmission efficiency of MCDV varies between male and female *G. nigrifrons* (9), and vector competence for MFSV varies within the same *G. nigrifrons* populations (5, 7). Although various hypotheses have been put forward to account for such variation (7, 10), the impact of a *G. nigrifons* virus on vector competence through direct pathogenic effects, or via interaction with MCDV or MFSV, remains to be assessed.

### Nucleotide sequence accession number. 

The genome sequence GNV.sqn GNV-1 was deposited in GenBank under the accession no. KP866792.
